# Supplementary material for: MetaRibo-Seq measures translation in microbiomes
Source: Nat Commun. 2020 Jun 29;11:3268. doi: 10.1038/s41467-020-17081-z (PMC7324362; doi:10.1038/s41467-020-17081-z)
Supplement: Supplementary file 10 — Supplementary Data 7 [file 41467_2020_17081_MOESM10_ESM.zip › File2/Confidence_VeryHigh_Taxonomy/132860_out.krona.html]

Javascript must be enabled to view this page.

members
magnitude
magnitudeUnassigned
count
unassigned
taxon
rank

132860\_out

19

2
superkingdom
19

1
phylum
1239

class
186801
1

1
order
186802

1
541000
family

genus
244127
1

1
species

SRS012273\_contig\_number\_contig-100\_25553.146659
1262700

203691
phylum
18

class
203692
18

18
order
136

18
family
137

genus
157
18

18
158
species

SRS013533\_contig\_number\_48280SRS014691\_contig\_number\_12574SRS016331\_contig\_number\_30736SRS018479\_contig\_number\_contig-100\_952.221302.221302SRS018826\_contig\_number\_36755SRS019026\_contig\_number\_contig-100\_3573.47578SRS019029\_contig\_number\_12757SRS020283\_contig\_number\_10469SRS023595\_contig\_number\_87932SRS047634\_contig\_number\_29885SRS049318\_contig\_number\_49768SRS063603\_contig\_number\_42589SRS077216\_contig\_number\_contig-100\_7356.7356SRS143068\_contig\_number\_contig-100\_53481.53482SRS144000\_contig\_number\_28225SRS148157\_contig\_number\_contig-100\_8153.8153SRS148193\_contig\_number\_37300SRS148284\_contig\_number\_contig-100\_7945.7945
